# The Intention to React to Sounds Induces Sleep Disturbances and Alters Brain Responses to Sounds during Sleep: A Pilot Study

**DOI:** 10.3390/clockssleep4040044

**Published:** 2022-10-19

**Authors:** Selina Ladina Combertaldi, Anna Zoé Wick, Björn Rasch

**Affiliations:** Division of Cognitive Biopsychology and Methods, Department of Psychology, University of Fribourg, Rue P.-A.-Faucigny 2, CH-1700 Fribourg, Switzerland

**Keywords:** on call, sleep, alarms, instructions, ERP, replication

## Abstract

Background: Pre-sleep intentions to react to stimuli during sleep affect sleep processes in spite of reductions in conscious awareness. Here, we compare influences of sounds presented during sleep (with and without intentions to react) with the effect of pre-sleep intentions on sleep (with and without sounds being present during sleep). Methods: Twenty-six young, healthy participants spent two experimental nights in the sleep laboratory. On one night, they were instructed to react to sounds during sleep (“on call”); on the other night, not (“neutral”). Unknown to the subjects, sounds were presented at a low volume in both nights in one group. No sound was presented in any of the two nights in the other group. Results: The instruction of being “on call” decreased objective sleep efficiency independently of sounds being present or not. In addition, event-related responses to sounds as well as slow-wave activity were reduced when being “on call”. Conclusions: Pre-sleep intentions to react impair sleep independently of sounds actually being present and influence brain responses to sounds during sleep. Our results highlight the importance of subjective relevance for reducing negative impact of external noise sources such as traffic or church bells.

## 1. Introduction

Sleep is beneficial for our mental and physical health [1,2,3] and plays a role in memory consolidation [4]. While sleep is a state of strongly reduced consciousness, acoustic stimuli are processed during sleep and can lead to arousals and awakenings when presented above a certain threshold [5]. During conscious wakefulness, we can voluntarily increase our ability to detect and perceive acoustic stimuli by shifting our attention to the relevant stimuli [6,7,8]. It is not fully clear whether attentional shifts are possible when consciousness is reduced. On one hand, automatic attentional shifts appear almost absent during sleep, as infrequent sounds in a strain of repetitive sounds (“Oddball” paradigm) do not evoke a clear mismatch negativity during non-rapid eye movement (NREM) sleep [9,10,11]. On the other hand, voluntary focusing of attention appears to be preserved during sleep: pre-sleep intentions to react to specific stimuli (e.g., the cry of one’s own child) typically lead to facilitated awakenings from sleep [12,13]. Similarly, knowing to be on call facilitates awakenings by specific sounds but can also lead to general sleep disturbances in many professions such as nursing or medical practice [14,15], firefighting [16,17], or engineering [18,19]. Importantly, Wuyts and colleagues [20] showed that even when no wakening sounds occurred during sleep, the pre-sleep instructions to be “on call” impaired objective sleep efficiency, as well as increased the time spent awake during the sleep period and increased beta-activity during sleep. However, no group that actually listened to the sounds during sleep was included in this study. Thus, the relative impact of the instructions on sleep quality, compared to the physical sounds, remains unknown.

Here, we aimed to (a) replicate Wuyts and colleagues’ [20] results and (b) directly compare the influence of pre-sleep intentions to a group that received the same instruction and actually received sounds during sleep. We propose that pre-sleep intentions to react to specific stimuli during sleep per se will affect the sleeping process independently of low-volume sounds being presented during sleep or not. In contrast, presentation of the same sounds without the instruction to react will cause no, or only minor, sleep impairments. To investigate whether the hypothesized differences in sleep architecture have behavioral consequences, psychomotor vigilance and memory retention across sleep were assessed.

**Figure 1:**
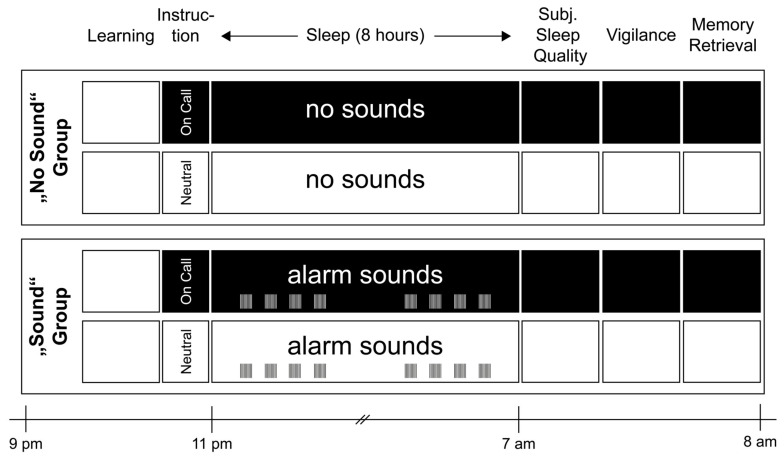
Procedure and experimental design. Participants slept in the sleep laboratory for one adaptation and two experimental nights. In the “on call” condition, participants were instructed to stay on call and react to auditory stimuli presented during the night by pressing a buzzer. The “neutral” instruction gave participants the permission to sleep regularly. Additionally, a between-subject condition was implemented: One group of 12 participants actually heard sounds during the night (“sound” group), whereas the other 14 participants had no sounds presented (“no sound” group). Sleep was recorded using PSG for objective sleep parameters. Subjective sleep parameters were asked in the morning in a questionnaire. In the evening, they performed a learning task of a semantic memory task, and its retrieval took place in the morning.

To test our hypothesis, 26 young and healthy participants spent two nights in the sleep laboratory with different instructions (see Figure 1 for an overview of the procedure): on one night, they were instructed to press a button whenever they heard a tone during sleep (“on call” condition). On the other night, no instruction to press a button was given (“neutral” condition). The nights occurred in a balanced order. Unknown to the participants, only a portion of the participants (*n* = 12) received tones during sleep during both nights (“sound” group). The other group (*n* = 14) received no sounds during any night of sleep (“no sound” group). We hypothesized that the pre-sleep instruction to react to tones would decrease sleep efficiency to a similar extent in both groups, independent of whether sounds were presented during sleep or not. In the “sound” group only, we predicted that the instructions to be “on call” would alter event-related potentials (ERPs) elicited by the sound compared to the neutral condition.

## 2. Results

### 2.1. Objective and Subjective Sleep Parameters

**Figure 2:**
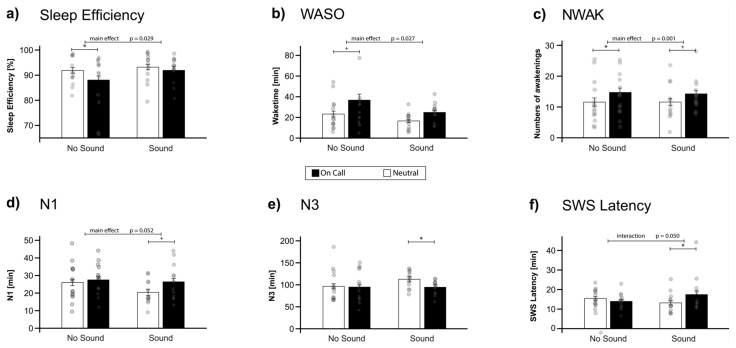
Objective sleep parameters. (**a**,**b**) Objective sleep efficiency was generally reduced by the pre-sleep instruction to be “on call” (main effect of instruction: *p* = 0.029). No interaction with sound presentation occurred (*p* > 0.90). As sleep onset latency was comparable between conditions and group, the reduction in sleep efficiency was mostly related to an increase in wake time after sleep onset (WASO), which showed a similar results pattern (main effect instruction: *p* = 0.27, interaction: *p* > 0.91). An exploratory post hoc *t*-test revealed that the effects of instruction were descriptively stronger in the “no sound” group. (**c**) The number of awakenings (NWAK) was significantly increased in both the “no sound” and “sound” group, independent of the presence of actual sounds during sleep (main effect instructions: *p* = 0.001, interaction: *p* > 0.76). (**d**,**e**) A main effect of instruction also occurred for N1 sleep, and a statistical trend, for SWS. Here, an exploratory *t*-test revealed a descriptively stronger effect on the sound group. (**f**) Only SWS latency was selectively influenced, supported by a significant interaction between instruction and sound (*p* = 0.007). Participants in the sound group spent longer when they were instructed to react to sounds, which was not the case in the no sound group. Means ± standard errors of the mean are indicated. Significant pair-wise comparisons from post hoc tests are indicated by +: *p* ≤ 0.1 *: *p* ≤ 0.05.

In accordance with our hypothesis, the instructions to be “on call” generally influenced objective sleep regardless of whether sounds were actually played during the night or not. First of all, objective sleep efficiency was significantly lower in the “on call” condition (89.84 ± 1.68%) as compared to the “neutral” condition (92.50 ± 1.11%; main effect of instruction: F (1,24) = 5.42, *p* = 0.029, η^2^ = 0.18)). In contrast, presentation of physical sounds during sleep did not generally impair sleep efficiency (“sound” group: 92.56 ± 1.10% vs. “no sound” group: 89.98 ± 1.68%; F (1,24) = 0.98, *p* = 0.333). Exploratory pair-wise comparisons revealed that the instructions significantly impaired sleep efficiency in the “no sound” group only (*p* = 0.042), but not in the “sound” group (*p* > 0.90). However, the interaction between the group (“sound” vs. “no sound” group) and instruction (“on call” vs. “neutral” condition) was not significant (F (1,24) = 1.24, *p* = 0.276, see Figure 2a, Table 1). Sleep onset latency (SOL) did not differ, neither between sound groups nor instruction (see Table 1). However, participants spent significantly more time awake during the night (WASO) when they received the instruction to be “on call” (31.23 ± 4.51 min) compared to “neutral” (20.17 ± 2.43 min), both in the “sound” group and in the “no sound” group (F (1,24) = 5.55, *p* = 0.027, η^2^ = 0.19); see Figure 2b. Furthermore, they woke up more often (number of awakenings, NWAK) when they were “on call” (14.50 ± 1.23 times) compared to the “neutral” condition (11.58 ± 1.24 times; F (1,24) = 14.32, *p* = 0.001, η^2^ = 0.37), again, independently of the presence of real sounds during sleep (see Figure 2c). The difference in number of awakenings induced by the instruction to be “on call” survived a correction for multiple comparisons (false discovery rate, FDR). Neither for time spent awake nor for the number of awakenings did we observe a main effect of sound group (“sound” vs. “no sound”) or any interaction (see Table 1). For subjective sleep parameters, we did not observe any significant influences of pre-sleep instructions or sound presentations during sleep (see Table 1).

The impact of pre-sleep instructions was also apparent on the level of sleep architecture, although the effects did not reach significance. Participants generally spent less time during slow-wave sleep in the “on call” condition (94.67 ± 5.43 min) compared to the “neutral” condition (103.92 ± 5.91 min). However, the effect only reached a statistical trend F (1,24) = 3.39, *p* = 0.078, η^2^ = 0.15). Similarly, participants spent more time in the light sleep stage N1 in the “on call” (27.00 ± 1.84 min) vs. “neutral” condition (23.44 ± 1.83 min) (F (1,24) = 4.16, *p* = 0.052). Other sleep stages (N2, REM sleep) were not affected by the instructions. Importantly, we did not observe any change in sleep stages with respect to the presentation of sounds (all main effects of sound group (“sound” vs. “no sound”) *p* > 0.30) and no interactions (all *p* > 0.11). However, exploratory pairwise comparisons revealed that the effects of instruction on sleep architecture were significant in the sound group (but not in the “no sound” group) for both SWS and N1 sleep (see Figure 2d–e). Interestingly, a significant interaction was observed for SWS latency (F (1,24) = 8.64, *p* = 0.007, η^2^ = 0.26): only when sounds were actually presented during sleep (“sound” group) did participants reach SWS significantly later when being “on call” (17.42 ± 1.91 min) compared to the “neutral” condition (13.17 ± 1.05 min, *p* = 0.014). In contrast, in the “no sound” group, participants reached SWS earlier when being “on call” vs. the “neutral” condition (13.93 ± 0.78 min vs. 15.43 ± 0.88 min, *p* > 0.78, see Figure 2f). For REM latency, neither a main effect nor the interaction reached significance (all *p* > 0.17).

### 2.2. The Effect of Instruction on Event-Related Potentials (ERP) during Sleep

**Figure 3:**
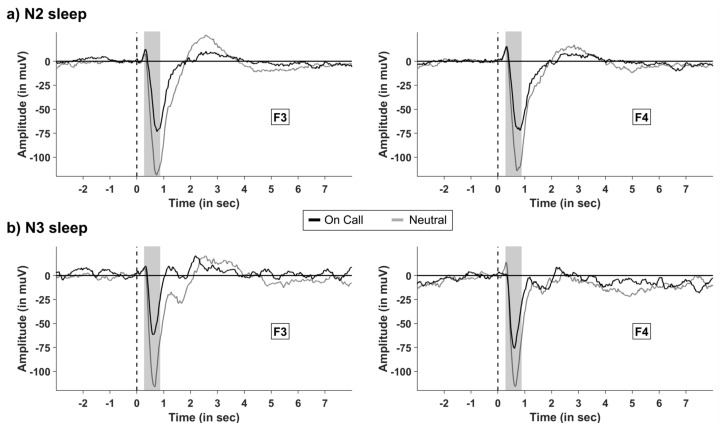
The effect of instruction on event-related potentials (ERP) in frontal electrodes during N2/N3 sleep. Grand-average ERP and reaction times to alarm sounds in the “sound” group in frontal electrodes F3 and F4 for N2 sleep (**a**) and N3 sleep (**b**). The instruction to react to sound stimuli consistently reduced the amplitude to sounds in a time range between 400 and 800 ms at both frontal electrodes recording sleep and both during N2 and N3 sleep. Data were baseline-corrected for a period of −1000 to 0 ms before the sound onset.

In the “sound” group only, we analyzed the effects on instructions on event-related potentials (ERPs) elicited by the sounds presented during sleep. Sound presentation during N2 and N3 sleep elicited the well-known K-complex-like response, with a large negative peak between 400 to 800 ms after the tone. The instruction to be “on call” significantly decreased the average amplitude in this time range: in N2 sleep, the amplitude in frontal electrodes (F3 and F4) was significantly lower in the “on call” condition (−43.71 ± 8.92 µV) compared with the ERPs to sounds in the “neutral” conditions (−84.66 ± 8.92 µV; *p* = 0.32, see Figure 3a). The same pattern occurred during N3 sleep (−55.92 ± 12.03 µV vs. −92.43 ± 9.95 µV, for “on call” and “neutral” conditions, respectively, *p* = 0.04; see Figure 3a–d). The effect of instructions was mainly seen in the frontal electrodes, as results revealed a significant interaction between the instruction (“on call” vs. “neutral”) and topography (frontal–central parietal–occipital, (F (1.47, 13.19) = 5.77, *p* = 0.022, η^2^ = 0.06). For no other result of the overall ANOVA including all eight electrodes using the factors instruction (“on call” vs. neutral), hemisphere (left vs. right), and sleep stage (N2 vs. N3) was a significant difference detected (all *p* > 0.08).

### 2.3. EEG Power Analysis

We conducted an EEG power analysis in accordance to the data reported by Wuyts et al. [20] by calculating the mean power during the combined sleep stage N2 and N3. We extracted the mean power of different frequency bands for both brain hemisphere separately. We chose a specific pair of electrodes for each frequency band (F3 and F4 for frontal slow oscillatory and slow-wave activity and frontal activity in the slow spindle band; C3 and C4 for central theta, alpha, and beta activity; P3 and P4 for parietal activity in the fast spindle band; see Section 4 for further details). Contrary to Wuyts et al. [20], we did not observe any significant result for the effect of the pre-sleep intention in the beta band (*p* > 0.80; see Table 2). However, power in the fast sleep spindle band was descriptively higher in the “no sound” group compared with the sound group, the main effect reached a statistical trend (F (1,24) = 3.84, *p* = 0.062, η^2^ = 0.12; (see Table 2).

### 2.4. Heart Rate and Heart Rate Variability

In addition to brain activity, we analyzed whether participants differed in physiological arousal during stimulation blocks depending on the instruction of being “on call” or not. We used three different indices provided by the analysis software Kubios HRV Premium 3.2.0 (Kubios Oy, Kuopio, Finland): a parasympathetic (PNS) and sympathetic nervous systems (SNS) tone index [22] as well as an index for heart rate variability (HRV triangular index). We focused our analysis on the stimulation blocks in the “sound” group. In the “no sound” group, arbitrary stimulation blocks were created (see Section 4). However, neither instruction nor sound presentations during sleep influenced the indices of heart rate and heart rate variability (all *p* > 0.41; see Table 1).

### 2.5. Memory Consolidation and Vigilance

We also tested whether the “on call” instruction as well as the presence of sounds during the night (“sound” vs. “no sound” group) affected memory consolidation or vigilance. Neither instruction (“on call” vs. “neutral”), sound group (“sound” vs. “no sound”), nor their interaction were significant (all *p* > 0.24). Encoding in the evening and retrieval in the morning did not differ (all *p* > 0.34). All results are presented in Table 1.

Vigilance was tested using a psychomotor vigilance test (PVT, [23]). Vigilance in the morning showed no significant effect (*p* > 0.73) or interaction, either for reaction time (*p* > 0.70), the numbers of reactions (*p* > 0.36), or errors (*p* > 0.22). For results, see Table 1.

## 3. Discussion

This study investigated the effect of pre-sleep intentions to react to auditory stimuli in the controlled setting of a sleep laboratory. We predicted that the instruction to be “on call” would alter sleep quality compared to a control condition, independently of sounds physically present during sleep or not. Our results confirmed this prediction and showed that the pre-sleep instruction to be “on call” impairs objective sleep efficiency and increases the time awake and number of awakenings during sleep. Thus, the instruction to be “on call” appears to result in a higher fragmentation of sleep. Furthermore, we observed statistical trends for more light sleep (N1) and less deep sleep (N3) in the “on call” condition.

Our results from the “no sound” group replicated the results reported previously by Wuyts and colleagues [20]: they also reported lower sleep efficiency and prolonged WASO after pre-sleep instruction to be “on call” without any sounds being played during sleep. In contrast, we were not able to replicate an increased sleep onset latency (SOL) and decrease subjective sleep quality ratings in the “on call” condition as reported by Wuyts et al. [20]. Furthermore, our results of the power analysis were opposite of those of Wuyts et al. [20], not showing an effect of pre-sleep instruction. Possible reasons are that participants in our study received the instruction directly before sleep when already lying in bed, whereas in the original study the instructions were presented 25 min before sleep. Furthermore, Wuyts’ instructions specified that no sound would be presented during the first 30 min after lights out. This instruction was left out in our “on call” instruction to see whether the instruction to be “on call” affected SOL or not.

In addition to the successful replication, we added a second group to the experimental design, which received sounds during sleep (“sound” group). Note that the study was double-blind; thus, neither the participants nor the experimenters knew whether sounds were presented or not. Importantly, the effects of pre-sleep instructions to be “on call” were similar in this group compared to the “no sound” group. Thus, listening to soft alarm sounds during sleep did not further increase the impairment of sleep. The presence of the pre-sleep instruction almost fully explained the sleep impairments. However, this finding might be due to our experimental setting in which we played sounds at soft volumes (ca. 42 dB). For these low-volume sounds, our results clearly show that the effect of the pre-sleep instruction to be “on call” is the main factor responsible for inducing sleep disturbances. However, louder sounds will clearly lead to a higher degree of sleep disturbances, whether they are associated with an intention to react or not.

Moreover, previous studies confirmed an association with stress for on-call work (for a review, see [24]). One possibility to measure physiological stress is physiological arousal [19,25,26,27]. Therefore, we investigated the heart rate variability (HRV) as well as indicators of the PNS and SNS activity. Neither the instruction nor the presence of sounds affected any of these arousal parameters, suggesting that the observed effects of pre-sleep intentions did not generalize to heart-related arousal indicators. Similarly, in real-life studies during on-call duty, physiological markers of stress (e.g., salivary cortisol) were not significantly altered [28,29]. In addition, in our study, there were no effects on vigilance in the morning or memory consolidation due to the “on call” instruction, in spite of significant disruptions in sleep measures.

### 3.1. Limitation

Our study has several limitations. First, the sample size would have to be substantially increased to improve statistical power. The current sample size is more in nature of a pilot study, and more subjects should be examined to draw clearer conclusions about the effects. In addition, most reported results did not survive a correction for multiple comparisons. Therefore, the reported results are preliminary and need to be replicated and confirmed in a pre-registered study using more rigorous hypotheses testing procedures.

Furthermore, the presented tones were rather quiet (ca. 42 dB). It is therefore possible that the signal during sleep was not particularly loud for participants. Sounds with higher volumes will clearly have a stronger impact on sleep and awakenings. In a next study, this should be taken into account, and alarm sounds should be chosen that are more distinct from environmental noise. Next, the sounds were presented in blocks (8 blocks of 10 sounds distributed over both night halves). Between two sounds within a block there was a pause of 1 min, during which the subject could fall asleep again. This time was very short. It might have been the case that when participants woke up in a block, all following sounds were heard in the waking state. In addition, we restricted our oscillatory power analysis to NREM sleep stages N2 and N3, as we expected the most relevant effects of pre-sleep intentions in these sleep stages. Future studies could extend this analysis to REM sleep. Finally, button presses could not be obtained and analyzed due to a failure in the recording software. Thus, we do not have a behavioral measure of the reactions executed after hearing the tone.

### 3.2. Outlook

The outstanding question is, how do the presented alarm sounds affect our brain during sleep? We assume that an attentional system is constantly monitoring and evaluating the importance of external sounds even during the sleep in spite of reduced conscious awareness state [30]. This attentional system can be influenced and “tuned” by pre-sleep intentions to react to specific stimuli during sleep, which leads to facilitated awakenings when the expected stimuli occur. However, as the intention generally increases monitoring efforts during sleep, pre-sleep intentions to react to stimuli will generally disturb sleep, as it probably remains active during sleep even in the absence of sounds. Such a sleep-monitoring system will share properties with our prospective memory system, which enables us to detect relevant stimuli and execute a certain action during wakefulness [31,32]. This is in line with the assumption that disturbed sleep is dependent on the cognitive state in the evening. If someone expects to be woken up not only because of on-call duty but also, for example, because of ringing church bells or a passing train during the night, the chances might be very high to do so. On the other hand, sleepers might benefit from the pre-sleep instruction “do not wake up because of the church bells ringing”. This seems to be an important aspect for adaptive sleep behavior and may increase chances of survival in dangerous surroundings, which remains interesting for future research.

Mechanistically, our results highlight the importance of our pre-sleep cognitions for sleep regulation and sleep quality, as we are able to influence the external monitoring of acoustic stimuli during sleep intentionally. In addition, our results support the notion that cognitive processes remain active (or a spontaneously reactivated) during sleep and can influence ongoing sleep processes [32,33]. Therefore, cognitive evaluation processes and cognitive content active during sleep need to be considered for evaluating sleep quality [34]. Future studies are needed to further understand the role and mechanisms underlying the influence of cognitive evaluations and re-activations during sleep on sleep disturbances, sleep fragmentation, and subjective sleep quality. Furthermore, real-life sleep studies need to examine whether sleep disturbances caused by external noises such as traffic or church bells also depend on our pre-sleep cognitions (e.g., subjective relevance we assign to these stimuli) and need to test whether reducing this relevance is a key factor for improving sleep quality.

In summary, we identified the pre-sleep intention as the major factor to explain sleep disturbances in “on call” situations, while the actual disturbance from sounds might play only a minor role. Our results have important consequences for real-life on-call situations that are known to impair sleep quality [24,29,35].

## 4. Materials and Methods

### 4.1. Methods

#### 4.1.1. Participants

In total, 26 young healthy students (17 women) with a mean age of 22.9 ± 3.0 (SD)) participated in the study. We randomly assigned 12 participants to the “sound” group, whereas 14 participants were included in the “no sound” group. Participants were recruited using an advertisement newsletter for students and among friends of the experimenter. They had no known sleep disorders (screened by the Pittsburgh Sleep Quality Index [36]). Participants were asked to keep a regular sleep rhythm and avoid night shifts. They were instructed to abstain from alcoholic and caffeinated beverages on the day before the experimental day and on the testing day. The Internal Review Board of the Department of Psychology at the University of Fribourg (protocol 2018–398; 11 July 2018), and all subjects gave written informed consent prior to participating. The study was conducted in accordance with the declaration of Helsinki. Subjects received either university course credits or monetary compensation of CHF 150 for their participation. Additionally, they were rewarded with CHF 20 for their efforts to react to sounds during the night. In case of an early drop out, payment was provided proportionately.

#### 4.1.2. Experimental Design and Procedure

The within-subject design included one adaptation and two experimental nights in the sleep laboratory at the University of Fribourg. In one of the experimental nights, we instructed participants to stay on call and react to auditory stimuli presented during the night (“on call” condition) by pressing a buzzer installed next to the pillow. To create a more realistic situation, the written instructions informed the participants to imagine that someone’s life is depending on their reaction time and that they get rewarded the faster their reaction to the stimuli is. The other night figured as a baseline (“neutral” condition), and participants slept regularly. The experimenter and the subjects were blind toward the type of instruction until the very last moment before sleeping. The order of the instructions was randomly assigned and balanced between subjects.

Additionally, we included a between-subject factor of a “sound” group. Each participant was randomly assigned to a “sound” or “no sound” group. In the “sound” group, during both experimental nights (“on call” and “neutral” condition), 80 sounds were presented each night. A speaker automatically presented eight blocks of 10 alarm sounds in the night. The setting ensured alarm sounds during the whole night. Sounds were presented using E-Prime (Psychology Software Tools Inc., Pittsburgh, PA, USA). The between-subject factor was double-blinded: neither participant nor experimenter knew about it until the end of data collection.

Each night, participants arrived at the sleep laboratory around 9 p.m. and filled out standardized questionnaires before the application of the polysomnography (PSG including electroencephalogram (EEG), electromyogram (EMG), electrooculogram (EOG), electrocardiogram (ECG)). The sleep schedule was standardized for all participants independent of their chronotype (see Figure 3). Afterward, they completed a memory task involving 80 semantically associated word pairs (PAL [37]), which is standard practice in our laboratory to see if there were effects on memory of impaired sleep. When lying in bed, they received the instruction for the night (“on call” or “neutral” condition). After 8 h of sleep, the experimenter woke the participants up by turning on the light. Directly after getting up, participants filled in standardized questionnaires (subjective sleep rating, mood), performed a 10 min psychomotor vigilance test (PVT, [23]), and completed the morning recall of the PAL. The session ended with removal of the PSG (see Figure 3).

### 4.2. Material

#### 4.2.1. On-Call Instruction

Instructions were given in a written form when participants were in bed and ready to sleep. Participants were instructed to be on call duty and to act as if they were responsible for another person, who would ring an alarm. The faster they reacted, the better, which would be compensated by receiving additional money for each correct reaction and losing money for each incorrect stimulus reaction. They were told that 10 to 100 alarm sounds would be presented at different volumes, and that it might be difficult to hear all of them. An example of this alarm sound was given.

#### 4.2.2. Sound Presentation during the Night

The experimenter started an E-Prime run (Psychology Software Tools Inc., Pittsburgh, PA, USA) after turning off the light. The file was started each night (“on call” or “neutral” condition). To ensure a double-blind setting, neither participants nor experimenters knew that there was a pre-programmed, between-subject-factor sound vs. no sound within the files. During the “on call” condition, sounds were presented block-wise using E-Prime (Psychology Software Tools Inc., Pittsburgh, PA, USA) for stimulus presentation. Directly after starting E-Prime, no sounds were presented for 30 min. Afterwards, sounds were presented in eight blocks. Each block included 10 sounds at 40–42 decibel volume. A break of 1 min allowed participants to fall back asleep between two alarm sounds. After 10 sounds (=one stimulus block) a 10 min break followed, where no sounds were presented. After the fourth block, a break of 2 h was inserted to provide sounds in both night halves (80 sounds in total, 4 blocks of 10 tones per night half). After finishing the presentation, E-Prime showed a black screen informing the experimenter that he/she could end the experiment by pressing any key.

During the neutral night, no sounds were presented. E-Prime presented a black page for 1 h before telling the experimenter to end the experiment by pressing any key.

#### 4.2.3. Sleep Setting

The participants slept in a quiet and dark environment in the sleep laboratory of the Cognitive Biopsychology and Methods Department at the University of Fribourg, Switzerland. The laboratory room corresponds to a room with standard furnishings (bed, bedside table, dresser, and desk) to have the environment as authentic as possible for the subjects and to grant a greater generalizability of the results to everyday life. A pressure switch (diameter about 1 cm) was fixed next to the pillow. Participants had to press this switch whenever they heard an alarm sound; therefore, it was fixed in one location, making it accessible to the participant at all times and not interfering with the sleep setting. The buzzer gave no feedback and made no noise when pressed. All participants spent the night in the same room to increase comparability between participants. The university building itself is located far from a main road, and noise from outside is negligible. Noise or video during the night was not recorded.

#### 4.2.4. Polysomnographic Recordings and Sleep Analysis

Sleep was measured using single gold-cap electrodes following the 10–20-EEG-system (F3, F4, C3, C4, P3, P4, O1, O2) with a sampling rate of 500 Hz. Impedances were kept below 5 kΩ. During recording, electrodes were referenced against Cz and re-referenced to the mastoids (M1 and M2). Additionally, we measured EMG, EOG, and ECG to complete the polysomnographic recording. Data were pre-processed with Brain Vision Analyzer 2.0 (Brain Products GmbH, Gilching, Germany), which filtered the data following the guidelines suggested by the American Association of Sleep Manual (AASM [38]). Two independent scorers, blind to the conditions, scored sleep in 30s periods following the AASM scoring setting [38]. One scoring was performed by a human scorer, and the second one was performed by The Siesta Group (The Siesta Group Schlafanalyse GmbH, Vienna, Austria). The two independent scorings were controlled by a third, independent scorer (human), who decided the final sleep stage in case of disagreements between the human scorer and the automatic scoring.

#### 4.2.5. Analysis of EEG Data

EEG data were pre-processed using BrainVision Analyzer 2.1 (Brain Products GmbH, Gilching, Germany). First, all data were filtered using a low-pass filter of 50 Hz and a high-pass filter of 0.1 Hz. A semi-automatic artefact correction was performed (pre-selecting artefacts of ±600 µV and an interval length of 200 ms), and remaining artefacts were removed manually. We calculated the average power of oscillatory activity in different frequency bands as follows [1,39,40]: slow-wave activity (SWA, 0.5–4.5 Hz), slow oscillatory activity (SO, 0.5-1 Hz), delta activity (1–4 Hz), theta activity (4.5–8 Hz), alpha activity (8–11 Hz), activity in the slow spindle (11–13 Hz), and fast spindle band (13–15 Hz), as well as beta activity (15–30 Hz). Data from lights-off in the evening to lights-on in the morning were analyzed and segmented for NREM sleep (N2 and N3 sleep) and REM sleep. Prior to data analyses, we conducted a conservative outlier correction (Mean ± 3 SD).

#### 4.2.6. EEG Power Analysis

For the frequency analysis, we used BrainVision Analyzer 2.1 (Brain-Products Inc, GmbH, Gilching, Germany). To conduct the Fast Fourier Transformation (FFT), we followed the procedure described by Ackermann and colleagues [41]. We set a high-pass (0.1 Hz) and a low-pass filter (40 Hz), and data were re-referenced to the average of both mastoid electrodes. Finally, an FFT with a 10% Hanning window and a resolution of 0.25 Hz was performed for every EEG channel to calculate the power in each frequency band. We analyzed segments from sleep onset to sleep offset (sleep without SOL), as well as sound blocks and arbitrary created blocks, at the same time, when no sounds were presented (sound presentation took place block-wise with a fixed starting and end point based on the start of E-Prime). Within these segments, sections of 2048 data points (4000 ms) with a 100-point overlap were created. An automatic artifact rejection excluded segments (exclusion criteria: EMG > 150 μV difference; EEG > 500 μV) before running the FFT. In the end, the power was averaged. For a more thorough analysis in RStudio (R Core Team, Vienna, Austria; RStudio Team, Boston, MA, USA), the mean power of all frequency bands was exported as mentioned in Section 4.2.5. More precisely, for each individual frequency band, a specific pair of electrodes was considered to calculate the mean power (F3 and F4: SO, SWA, slow spindle band; C3 and C4: theta, alpha, and beta; P3 and P4: fast spindle band).

#### 4.2.7. Event-Related Potentials (ERP) Analysis during Sleep

Event-related potentials (ERP) reflect neurophysiologic activity without the participant’s awareness for stimuli during sleep [39,40]. ERPs were analyzed using BrainVision Analyzer 2.1 (Brain Products GmbH, Gilching, Germany). Only sounds presented during artefact-free NREM sleep stages N2 and N3 were included in the analysis. Segments included the signal from 3000 ms before the tone until 8000 ms after the tone. The signal was baseline corrected using 1000 ms before the onset of the tone. For the analyses, the mean potential between 400 ms to 800 ms after the sound presentation was extracted and further analyzed using “R”. ERPs were plotted in Matlab (Mathworks, Natick, MA, USA).

#### 4.2.8. Analysis of ECG and Physiological Arousal

For the analysis of ECG, we used Kubios HRV Premium 3.2.0 (Kubios Oy, Kuopio, Finland). Therefore, the ECG signal for the whole night (lights-off to lights-on) was exported in EDF+-format using BrainVision Analyzer Version 2.0 (Brain Products GmbH, Gilching, Germany). Kubios HRV Premium offers an automatic artifact correction based on RR series to eliminate ectopic beats and artifacts on unfiltered data [22]. Afterward, data were analyzed in time and frequency domain and used for calculation the mean heart rate (mean HR, in beats per minutes (bpm)) and mean RR-interval (mean RR, in milliseconds) as an index for physiological arousal [42]. Additionally, we used the HRV triangular index and the PNS, SNS, and stress indices provided by Kubios to evaluate an index for physiological stress. PNS index is a parasympathetic nervous system (PNS) tone index based on mean RR, whereas in the SNS index, the sympathetic nervous system (SNS) reflects based on mean HR [22].

#### 4.2.9. Questionnaires

In the first night, participants filled out a general questionnaire for personal information, the Edinburgh Inventory for Handedness [43], the Pittsburgh Sleep Questionnaire Index [36], and a questionnaire for chronotype [44]. Additionally, in each session, participants filled out a general questionnaire and a questionnaire on mood [45]. In the morning, participants were asked to rate their subjective sleep quality [21] and again their mood [45]. All questionnaires were presented online using SoSci Survey [46].

#### 4.2.10. Memory Measurement

Episodic memory was tested with a paired-associated learning task [37]. Each evening, the participants learned a list of 80 semantically related word pairs [37,47]. Each trial started with the first word of a pair, which was presented for 3000 ms and followed by a 500 ms blank interval, which separated the single trials. The words were presented in black font on a white screen via E-Prime (Psychology Software Tools Inc., Pittsburgh, PA, USA). Every pair was presented only once, while the order was kept constant. Immediately after learning, participants were confronted with a cued recall test. Here, they had to come up with the corresponding word when the first word was displayed. During the recall test, the word pairs were presented for an infinite amount of time or until the participant pressed enter. Right after, the correct answer was presented for 1000 ms, followed by a 500 ms blank interval which separated the single trials. After each word pair was tested once and provided the participant gave the correct answers, a second recall test followed. The participants did not receive any correction, and right after filling in the answer, a 500 ms blank interval followed. During recall, the order of the word pairs differed from learning phase but was kept constant across subjects. A second recall phase took place in the morning. Performance was measured as percentage of words recalled in the morning relative to the amount remembered immediately after learning. Response time was not restricted.

#### 4.2.11. Psychomotor Vigilance Test (PVT)

In the morning, participants performed the psychomotor vigilance test (PVT, [23]). For 10 min, subjects were asked to press the space key with their non-dominant hand as soon as they recognized the millisecond counter on the screen, which appeared at random intervals. After the keypress, the reaction time in milliseconds was shown for 1000 ms, then the counter was zeroed, and this was repeated.

#### 4.2.12. Statistical Analysis

Data were analyzed using a repeated measurement analysis of variance (ANOVA) with the within-subject factor instruction (“on call” vs. “neutral”) and the between-subject factor sound group (“sound” vs. “no sound” group). According to sleep quality, our main outcome variables were subjective and objective sleep quality, sleep onset latency (SOL), waketime after sleep onset (WASO), number of awakenings during sleep (NWAK) and sleep depth. Total sleep length was excluded from this primary analysis as participants were awakened 8 h after lights-off. In the exploratory analysis, we additionally analyzed further sleep parameters and oscillatory power during NREM sleep as well as vigilance, sleep-associated memory consolidation, and heart rate variability (HRV).

In addition, for the primary outcome variables, a factor type of parameter (“subjective” vs. “objective” parameters) was used. Exploratory post hoc paired *t*-tests were conducted to examine the result pattern of interaction effects. We set the level of significance to *p* = 0.05 and report effect sizes (η^2^) only for significant data. Data were analyzed using R Studio (R Core Team, Vienna, Austria; RStudio Team, Boston, MA, USA). Results are presented as means ± standard errors. As soon as the sphericity was violated, we applied the Greenhouse–Geisser correction.

## 5. Conclusions

To summarize, our results replicate the findings of Wuyts et al. [20], showing that pre-sleep instructions to be “on call” impair objective sleep quality. In addition, we extended their result by showing that the impairing effects of pre-sleep intentions to react to specific stimuli are comparable with a group that actually heard sounds during sleep.

## Figures and Tables

**Table 1:** Objective and subjective sleep parameters.

	“No Sound” Group						“Sound” Group											
	Neutral			On Call			Neutral			On Call			Pre-Sleep Intention		Sound Group		Interaction	
	Mean	±	SEM	Mean	±	SEM	Mean	±	SEM	Mean	±	SEM	F-Value	*p*-Value	F-Value	*p*-Value	F-Value	*p*-Value
**Objective Sleep Parameters**																		
**Sleep Efficiency (%)**	91.90	±	1.11	88.06	±	1.68	93.20	±	1.11	91.91	±	1.68	**5.42**	**0.029**	0.98	0.333	1.24	0.276
**SOL (min)**	22.82	±	4.36	22.11	±	3.50	21.96	±	4.80	21.96	±	4.54	0.01	0.905	0.00	0.950	0.01	0.912
**WASO (min)**	23.25	±	2.89	36.71	±	5.80	16.58	±	1.59	24.83	±	1.83	**5.55**	**0.027**	2.96	0.098	0.31	0.585
**NWAK**	11.57	±	1.34	14.71	±	1.41	11.58	±	1.17	14.25	±	1.04	**14.32**	0.001 *	0.01	0.926	0.09	0.761
**N1 (min)**	26.04	±	2.07	27.50	±	1.75	20.42	±	1.33	26.42	±	2.00	4.16	0.052	1.09	0.307	1.68	0.207
**N2 (min)**	212.71	±	6.51	203.71	±	7.37	213.08	±	4.91	219.42	±	6.25	0.14	0.716	0.47	0.948	2.15	0.156
**N3 (min)**	96.50	±	7.02	94.86	±	6.94	112.58	±	3.91	94.46	±	3.20	3.39	0.078	0.59	0.450	2.68	0.115
**REM (min)**	100.50	±	3.91	93.36	±	3.80	95.17	±	2.84	92.88	±	4.21	2.10	0.160	0.19	0.668	0.51	0.481
**SWS Latency (min)**	15.43	±	0.88	13.93	±	0.78	13.17	±	1.05	17.42	±	1.91	1.40	0.248	0.08	0.784	**8.64**	**0.007**
**REM Latency (min)**	86.46	±	4.81	84.21	±	4.95	99.88	±	8.43	78.88	±	5.10	2.00	0.170	0.20	0.659	1.47	0.237
**TST (min)**	459.00	±	5.99	456.18	±	3.48	457.83	±	4.75	458.04	±	4.46	0.12	0.730	0.00	0.960	0.14	0.710
**N1 (%)**	5.69	±	0.46	6.02	±	0.37	4.48	±	0.30	5.77	±	0.42	**4.25**	**0.050**	1.11	0.303	1.63	0.214
**N2 (%)**	46.49	±	1.51	44.55	±	1.50	46.50	±	0.86	47.84	±	1.15	0.13	0.723	0.50	0.485	1.92	0.179
**N3 (%)**	20.96	±	1.46	20.78	±	1.51	24.59	±	0.82	20.70	±	0.75	2.93	0.100	0.67	0.421	2.81	0.107
**REM (%)**	21.86	±	0.76	20.47	±	0.81	20.76	±	0.54	20.28	±	0.90	1.56	0.223	0.24	0.630	0.35	0.561
**WASO (%)**	5.02	±	0.60	8.18	±	1.34	3.68	±	0.38	5.43	±	0.42	**5.85**	**0.024**	0.67	0.115	0.46	0.505
**Subjective Sleep Parameters**																		
**Sleep Quality**	3.30	±	0.16	3.34	±	0.12	3.74	±	0.16	3.19	±	0.12	1.71	0.204	0.41	0.528	2.65	0.117
**SOL (min)**	22.14	±	2.54	20.00	±	2.21	17.58	±	2.64	17.92	±	2.85	0.17	0.685	0.54	0.468	0.26	0.617
**WASO (min)**	11.00	±	2.28	15.93	±	3.44	10.83	±	2.97	13.58	±	3.29	1.61	0.216	0.06	0.811	0.12	0.728
**NWAK**	2.36	±	0.27	2.21	±	0.27	2.25	±	0.31	3.17	±	0.20	1.03	0.320	1.04	0.318	2.40	0.135
**Sleep Depth**	3.14	±	0.22	3.14	±	0.23	3.75	±	0.22	3.33	±	0.21	0.54	0.470	1.27	0.271	0.63	0.436
**Memory**																		
**Learning**	55.36	±	3.35	54.64	±	2.75	58.75	±	2.60	59.67	±	2.82	0.00	0.981	0.56	0.461	0.27	0.608
**Recall**	53.71	±	3.36	52.36	±	3.04	57.58	±	2.49	58.58	±	3.02	0.03	0.864	0.75	0.395	0.57	0.459
**Consolidation (%)**	97.05	±	0.95	94.92	±	1.14	98.21	±	0.83	97.71	±	0.95	1.47	0.230	1.52	0.229	0.51	0.483
**Vigilance**																		
**Mean RT (ms)**	328.73	±	6.96	332.84	±	5.90	327.82	±	5.92	326.83	±	6.61	0.11	0.738	0.09	0.771	0.27	0.609
**Errors**	0.77	±	0.15	1.08	±	0.33	0.67	±	0.16	1.33	±	0.37	1.59	0.220	0.04	0.840	0.22	0.642
**Reactions**	76.69	±	0.39	77.54	±	0.66	76.75	±	0.65	76.58	±	0.67	0.18	0.675	0.28	0.604	0.36	0.556
**Physiological Arousal**																		
PNS index	1.30	±	0.40	1.25	±	0.40	1.60	±	0.30	1.34	±	0.34	0.68	0.418	0.11	0.741	0.32	0.577
SNS index	−0.51	±	0.24	−0.45	±	0.21	−0.48	±	0.33	−0.43	±	0.27	0.06	0.809	0.00	0.958	0.00	0.986
HRV triangular index	11.45	±	0.90	11.94	±	0.98	13.21	±	1.04	12.18	±	1.00	0.29	0.593	0.45	0.507	2.34	0.139

Notes. Objective values are based on polysomnographic recordings. Non-rapid eye movement (NREM) sleep; stage 1, 2, and 3 sleep (N1, N2, N3); rapid eye movement sleep (REM); waketime after sleep onset (WASO); number of awakenings (NWAK); total sleep time (TST); sleep onset latency (SOL); slow-wave sleep latency (SWS latency); and REM sleep latency (REM latency) are all measured in minutes (min), and the percentages indicate parietal percentage of TST (%). Three participants in the no sound group spent more than 50 min awake (2 in the on call condition, 1, in the neutral condition; all other participants were awake for less than 40 min. Subjective parameters are based on subjective ratings in the SF-A-R [21]. For memory, numbers indicate absolute or relative values of correctly recalled words that were presented in the evening (learning phase with first recall) and in the morning (retrieval phase with second recall). Consolidation refers to the difference in performance between learning and retrieval phases. For vigilance, the reaction time (RT), the number of reactions, and number of errors during the 10 min of the psychomotor vigilance task (PVT) were measured. As an objective measurement of arousal, Kubios (Kubios Oy, Kuopio, Finland) provided an index for the parasympathetic nervous system (PNS index; based on mean RR interval in ms), sympathetic nervous system (SNS index; based on mean HR in beats per minute (bpm)), and heart rate variability (HRV triangular index). Values are means ± standard error of mean (SEM). *: Significant (*p* < 0.05) after correction for multiple comparisons using the false discovery rate (FDR). *p*-values in bold are significant (*p* ≤ 0.05) in a single test without correction.

**Table 2:** Results of the EEG Power Analysis during N2 + N3 sleep.

	“No Sound” Group						“Sound” Group											
	Neutral			On Call			Neutral			On Call			Pre-Sleep Intention		Sound Group		Interaction	
	Mean	±	SEM	Mean	±	SEM	Mean	±	SEM	Mean	±	SEM	F-Value	*p*-Value	F-Value	*p*-Value	F-Value	*p*-Value
**Left**																		
**Delta**	92.36	±	0.89	92.53	±	0.91	93.19	±	0.60	93.53	±	0.68	0.50	0.485	0.71	0.409	0.06	0.814
**Theta**	4.49		0.39	4.54		0.42	4.42		0.52	3.87		0.45	1.29	0.266	0.39	0.538	1.92	0.179
**Alpha**	1.77	±	0.18	1.75	±	0.20	1.83	±	0.21	1.73	±	0.27	0.37	0.549	0.01	0.934	0.18	0.675
**Slow Spindles**	1.12	±	0.20	0.94	±	0.15	0.80	±	0.10	0.77	±	0.10	1.61	0.216	1.53	0.228	0.74	0.397
**Fast Spindles**	1.39	±	0.23	1.35	±	0.22	0.94	±	0.10	0.88	±	0.12	0.21	0.650	3.84	0.062	0.00	0.951
**Beta**	0.59	±	0.08	0.60	±	0.09	0.49	±	0.06	0.48	±	0.06	0.02	0.894	1.15	0.295	0.15	0.705
**Right**																		
**Delta**	92.50	±	0.96	93.32	±	0.83	92.88	±	0.51	93.31	±	0.59	1.73	0.201	0.03	0.856	0.16	0.691
**Theta**	4.50	±	0.41	4.46	±	0.43	0.49	±	0.49	3.95	±	0.42	1.65	0.211	0.22	0.647	1.14	0.297
**Alpha**	1.77	±	0.19	1.72	±	0.22	0.20	±	0.20	1.74	±	0.26	0.42	0.525	0.01	0.917	0.03	0.859
**Slow Spindles**	0.94	±	0.16	0.92	±	0.16	0.82	±	0.09	0.79	±	0.09	0.20	0.659	0.47	0.501	0.00	0.967
**Fast Spindles**	1.20	±	0.23	1.12	±	0.19	0.86	±	0.08	0.81	±	0.11	0.43	0.517	2.18	0.152	0.02	0.895
**Beta**	0.58	±	0.09	0.57	±	0.08	0.48	±	0.05	0.48	±	0.05	0.06	0.815	0.98	0.332	0.01	0.912

Notes. EEG Power Analysis. Mean power was calculated in percentage during the combined sleep stages N2 and N3. Different frequency bands were derived from the most characteristic areas (frontal electrodes for delta and slow spindles; central electrodes for theta, alpha, and beta; parietal electrodes for fast spindles). Values are means ± standard error of mean (SEM).

## Data Availability

Datasets analyzed during the current study are available online on https://osf.io.
